# Paraneoplastic necrotizing myopathy associated with adenocarcinoma of the lung – a rare entity with atypical onset: a case report

**DOI:** 10.1186/1752-1947-7-112

**Published:** 2013-04-25

**Authors:** Angela Acciavatti, Tiziana Avolio, Simone Rappuoli, Luca Foderi, Vittoria Soldati, Massimo Franchi, Nila Volpi, Ranuccio Nuti

**Affiliations:** 1 Department of Internal Medicine, University of Siena, Viale Bracci, 53100, Siena, Italy

**Keywords:** Paraneoplastic myopathy, Paraneoplastic necrotizing myopathy, Paraneoplastic syndrome

## Abstract

**Introduction:**

Inflammatory myopathies (such as dermatomyositis and polymyositis) are well-recognized paraneoplastic syndromes. However, paraneoplastic necrotizing myopathy is a more recently defined clinical entity, characterized by rapidly progressive, symmetrical, predominantly proximal muscle weakness with severe disability, and associated with a marked increase in serum muscle enzyme levels. Paraneoplastic necrotizing myopathy requires muscle biopsy for diagnosis, which typically shows massive necrosis of muscle fibers with limited or absent inflammatory infiltrates.

**Case presentation:**

We report the case of an 82-year-old Italian-born Caucasian man who was admitted to hospital because of heart failure and two drop attacks. Over the following days, he developed progressive severe weakness, dysphagia, and dysphonia. Testing showed increasing serum muscle enzyme levels. Electromyography showed irritative myopathy of the proximal muscles and sensorimotor polyneuropathy. Muscle biopsy (left vastus lateralis) showed massive necrosis of muscle fibers with negligible inflammatory infiltrates, complement membrane attack complex deposition on endomysial capillaries, and moderate upregulation of major histocompatibility complex-I. Computed tomography of the thorax showed a nodular mass in the apex of the right lung. The patient was diagnosed with paraneoplastic necrotizing myopathy. In spite of high-dose corticoid therapy, he died 1 month later because of his aggressive cancer. Subsequent electron microscopic examination of a muscle biopsy specimen showed thickened walls and typical pipestem changes of the endomysial capillaries, with swollen endothelial cells. Poorly differentiated adenocarcinoma of the lung was confirmed on post-mortem histological examination.

**Conclusions:**

Paraneoplastic necrotizing myopathy is a rare syndrome with outcomes ranging from fast progression to complete recovery. Treatment with corticosteroids is often ineffective, and prognosis depends mainly on the characteristics of the underlying cancer. This case shows that paraneoplastic necrotizing myopathy may have an atypical appearance, and should be considered in elderly patients with neoplastic disease. In this case, the diagnosis was delayed by the unusual clinical picture that suggested heart disease rather than muscle disease.

## Introduction

Paraneoplastic necrotizing myopathy is a rare condition associated with cancer and characterized by massive necrosis of muscle fibers in the absence of significant inflammation [1-11]. It is part of the distinct disease entity of necrotizing myopathy, which has been added as an independent new domain to idiopathic inflammatory myopathy [6,7]. As autoimmunity is considered to play a role in the development of this condition, it is currently known as necrotizing autoimmune myopathy (NAM) [12]. An association between myopathy and cancer has been reported for the past century. This myopathy occurs in less than 1% of patients with cancer, and often precedes diagnosis of the cancer itself. Paraneoplastic necrotizing myopathy differs from primary myositis in that the prognosis is dependent on the underlying malignancy. Diagnosis of the underlying cancer is therefore crucial to the management of these patients. Paraneoplastic myopathy presents with symmetrical proximal muscle weakness, with acute or subacute onset of other symptoms such as dysphagia. Compared with non-neoplastic myopathy, paraneoplastic myopathy is much more likely to be associated with a marked increase in serum creatine kinase (CK) level and electromyographic changes characteristic of irritative myopathy [1]. This condition is associated with many neoplasms, including gastrointestinal adenocarcinoma, non-small cell lung carcinoma, and breast cancer.

Paraneoplastic necrotizing myopathy is diagnosed on histological examination of biopsy specimens, and is characterized by massive necrosis of muscle fibers and very few inflammatory changes. Some cases have focal or general capillary depletion and complement membrane attack complex (MAC) deposition in a significant proportion of endomysial capillaries [2,3,12]. Thickened and hyalinized capillaries, called pipestem capillaries, are an ultrastructural finding associated with necrotizing myopathy [2,3,7] which has also been described in paraneoplastic cases [2]. The minimal inflammatory infiltrate allows necrotizing myopathy to be distinguished from other cancer-related myopathies [4].

Here we report the case of a man who presented with heart failure and elevation of serum muscle enzyme levels; he was diagnosed with paraneoplastic necrotizing myopathy. He had a poorly differentiated lung adenocarcinoma, which had not previously been identified. This case highlights the difficulty of diagnosing paraneoplastic necrotizing myopathy, which may be underreported.

## Case presentation

An 82-year-old Italian-born Caucasian man was admitted to hospital because of heart failure and two falls without loss of consciousness the day before. On arrival, he was dyspneic with an oxygen saturation of 80% on room air. He had minor bruising on his chin and left elbow because of the falls.

**Figure 1:**
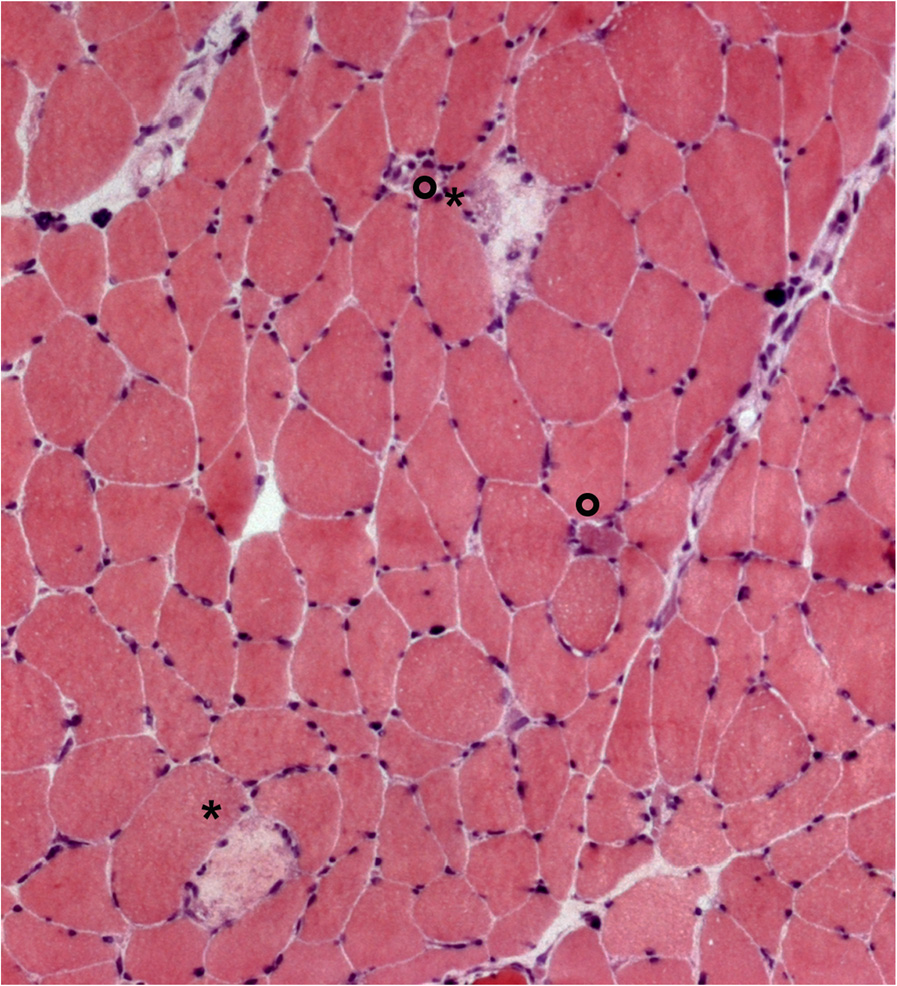
**Vastus lateralis muscle biopsy.** Myopathic changes with increased variability of muscle fiber diameter and sporadic centralized nuclei. Necrotic fibers (*) and regenerating fibers with basophilic cytoplasm (°). Hematoxylin and eosin staining, ×100.

**Figure 2:**
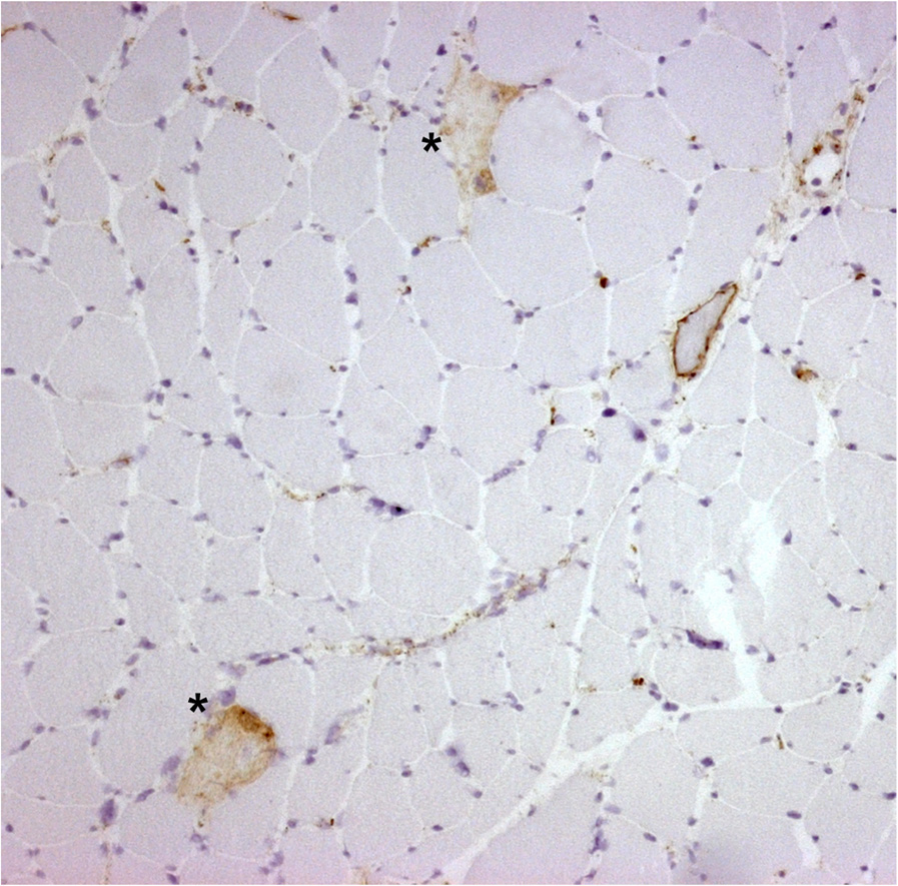
**Vastus lateralis muscle biopsy.** Immunohistochemical staining for complement membrane attack complex (MAC) on a section adjacent to that shown in Figure 1. The necrotic fibers (*) show MAC deposition in the membrane and cytoplasm. MAC: immunoperoxidase staining, ×100.

**Figure 3:**
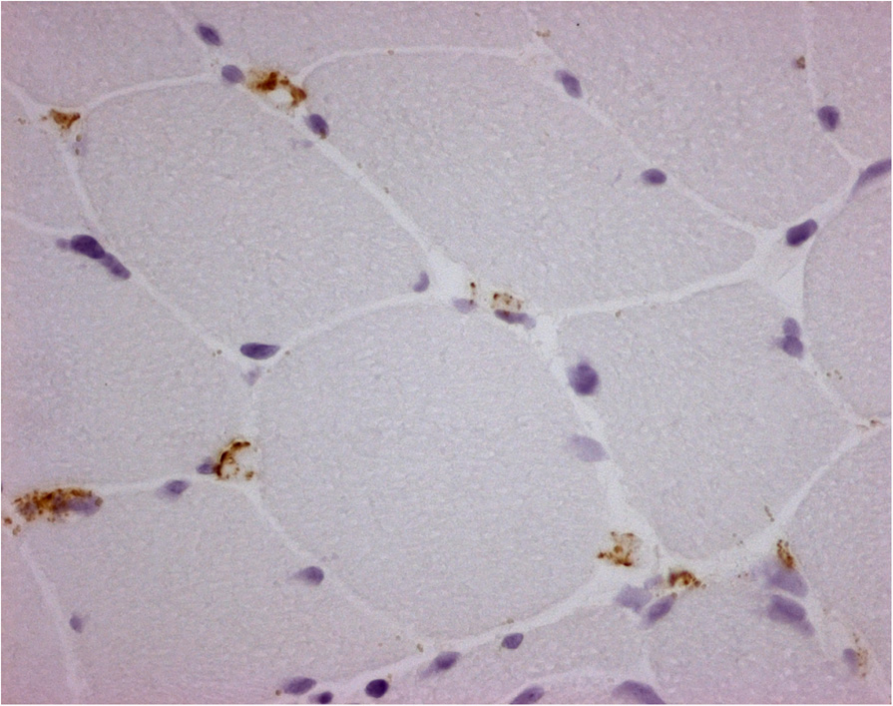
**Vastus lateralis muscle biopsy.** Endomysial capillaries with complement membrane attack complex (MAC) deposition. MAC: immunoperoxidase staining, ×400.

**Figure 4:**
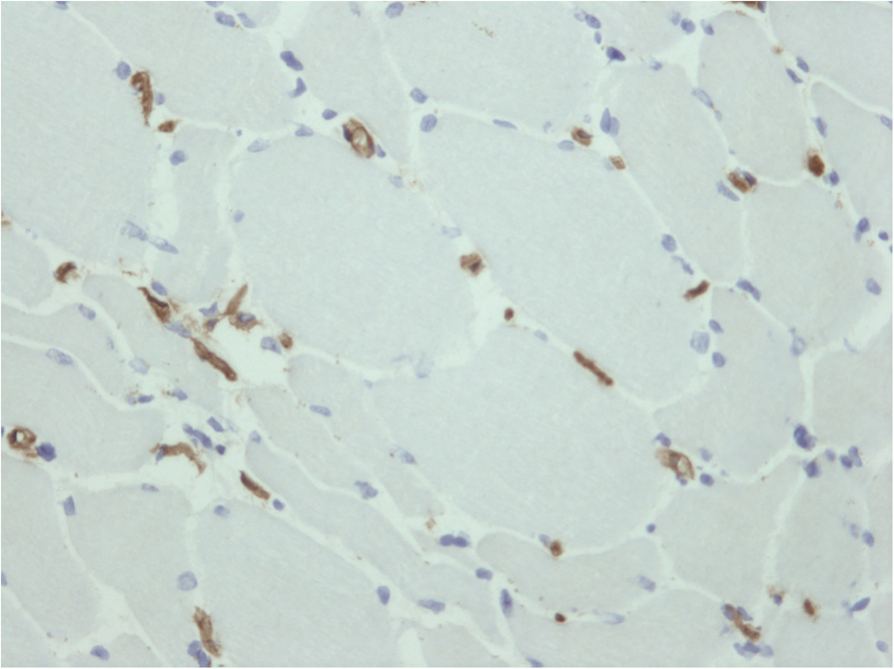
**Vastus lateralis muscle biopsy.** Lowered density of endomysial capillaries: several fibers have no capillaries in the endomysial space (number of capillaries/mm^2^ of cross-sectional area: 148.2; normal value 393.2). CD31: immunoperoxidase staining, ×200.

**Figure 5:**
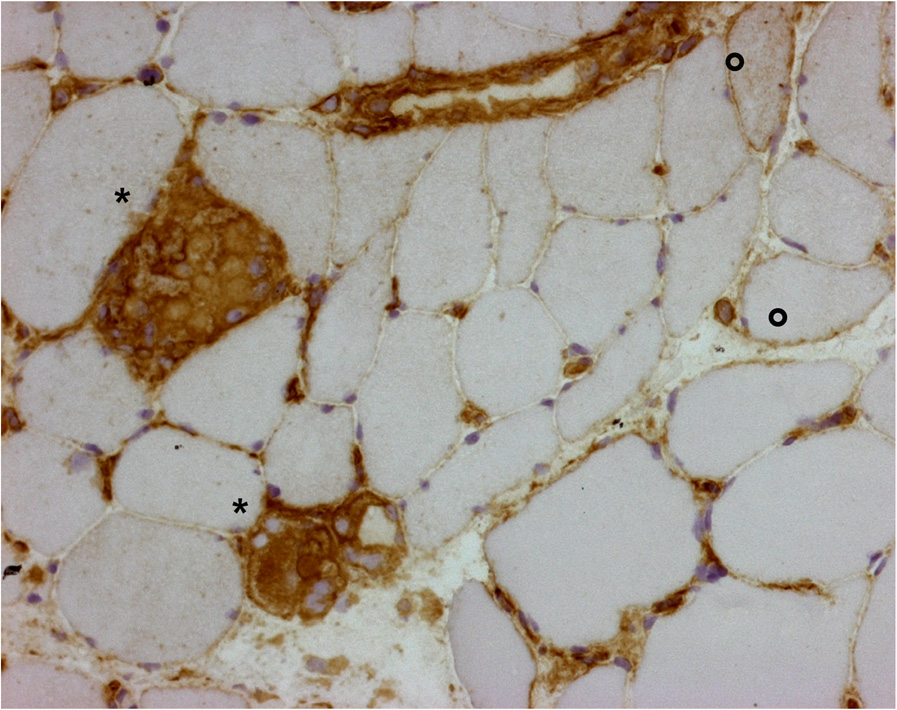
**Vastus lateralis muscle biopsy.** Necrotic fibers (*) with macrophage invasion show strong major histocompatibility complex (MHC)-I expression. Global MHC-I upregulation is very mild, with neoexpression on a limited subset of intact fibers (°). MHC-I: immunoperoxidase staining, ×200.

He had a past history of myocardial infarction, hypertension, and chronic atrial fibrillation. His regular medications were diuretics, aspirin, angiotensin-converting enzyme inhibitors, and amiodarone. Physical examination showed that he was tachycardic and dyspneic at rest, with cyanotic lips. Coarse crackles were heard throughout both lung fields. A chest X-ray showed cardiomegaly and pulmonary edema. Arterial blood gas analysis showed pH 7.40, partial pressure of carbon dioxide 37.2mmHg, partial pressure of oxygen 45.9mmHg, and bicarbonate 23.4mmol/L. Electrocardiography showed atrial fibrillation and widespread ST depression. Laboratory tests showed elevated serum levels of CK (5308IU/L), myoglobin (3000ng/mL), and lactate dehydrogenase (81,955IU/L). Serum alanine and aspartate aminotransferase levels were also elevated, but MB-CK and troponin T levels were in the normal range. Echocardiography showed post-ischemic dilated cardiomyopathy resulting from his previous anteroseptal myocardial infarction. He was treated with intravenous furosemide, nitrates, and supplemental oxygen by simple face mask. His respiratory status improved slightly, his heart failure resolved, and his oxygen saturation slowly normalized. Five days later, his serum muscle enzyme levels were still elevated (CK >3000IU/L, myoglobin >3000ng/mL, and aldolase 47.2IU/L) and he complained of progressive muscle weakness (Medical Research Council grade 2-3/5), dysphonia, and eventually dysphagia. A neurological examination showed symmetrical severe proximal muscle weakness (grade 2/5 for hip flexion and extension, grade 2/5 for shoulder abduction and elbow flexion and extension). Electromyography showed fibrillations, positive sharp waves, and brief polyphasic motor unit potentials, mainly in the proximal muscles, associated with sensorimotor polyneuropathy. Slowed latency and reduced amplitude were detected in the tibial and sural nerves (1.8mV and 4.0μV, respectively). Motor nerve conduction velocity was 40.3m/s in the tibial nerves and 38.5m/s in the sural nerves. Because of these clinical and biochemical signs of myolysis, immunodiagnostic tests for inflammatory myopathies were performed. Antinuclear antibody was negative by indirect immunofluorescence but was positive on human epithelial cell type 2 cells (1:640), anti-extractable nuclear antigen antibody (immunoblotting) was positive for SSA and for two unknown bands (65 and 74kD), and anti-Jo1 was negative. Tumor markers were positive (carcinoembryonic antigen, alpha-fetoprotein, CA-125, and CA 15–3). Testing for viral antibodies was negative (cytomegalovirus, herpes virus, adenovirus, Coxsackie virus, echo virus, and hepatitis B and C virus). Complement and immunoglobulin levels were normal. Ten days later, a vastus lateralis muscle biopsy was performed. Immunohistological examination showed scattered necrotic and regenerating muscle fibers, minimal cellular infiltration, slightly increased endomysial connective tissue, no perifascicular atrophy, moderate upregulation of major histocompatibility complex (MHC)-I with neoexpression on some non-necrotic fibers, multifocal MAC deposition in endomysial capillaries, and capillary loss (Figures 1, 2, 3, 4, and 5). These features were consistent with necrotizing myopathy. Mild fiber type grouping, suggestive of a collateral reinnervation process, was also detected.

**Figure 6:**
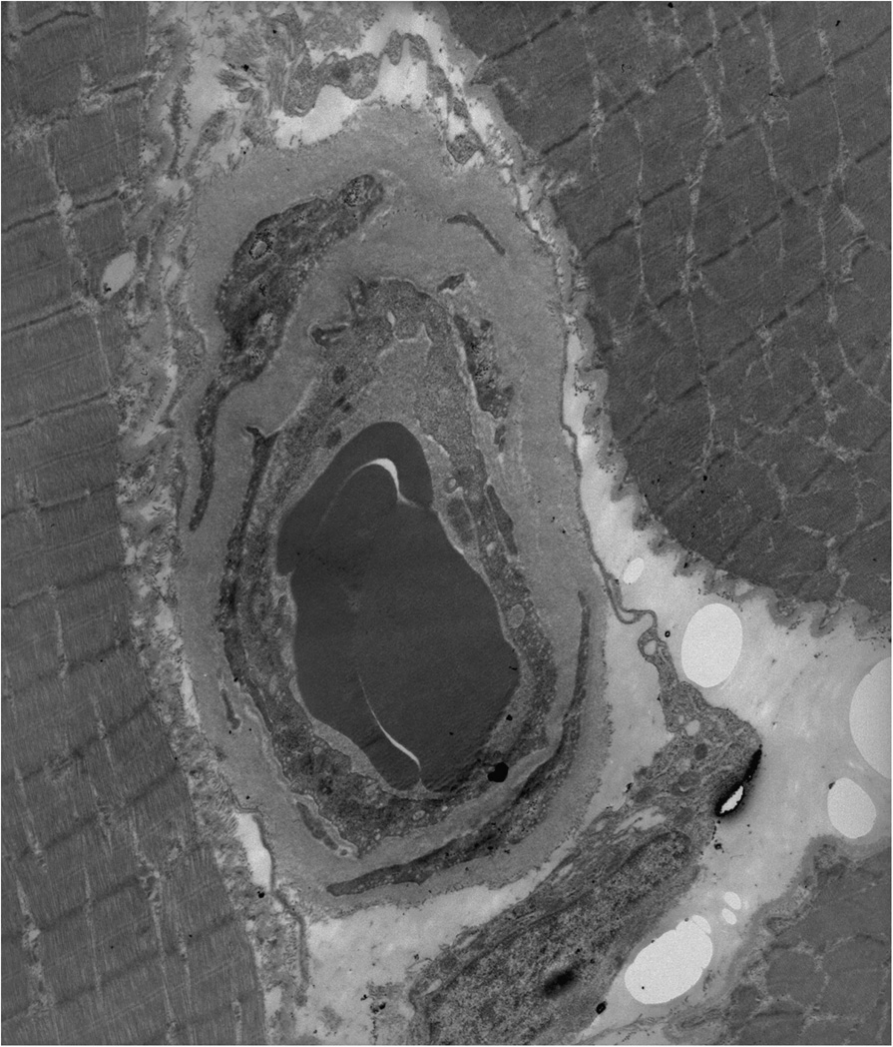
**Vastus lateralis muscle biopsy.** Thickened endomysial capillary wall, surrounded by a thick layer of amorphous material including the pericytes, and collagen fibers. Transmission electron microscopy, ×5200.

**Figure 7:**
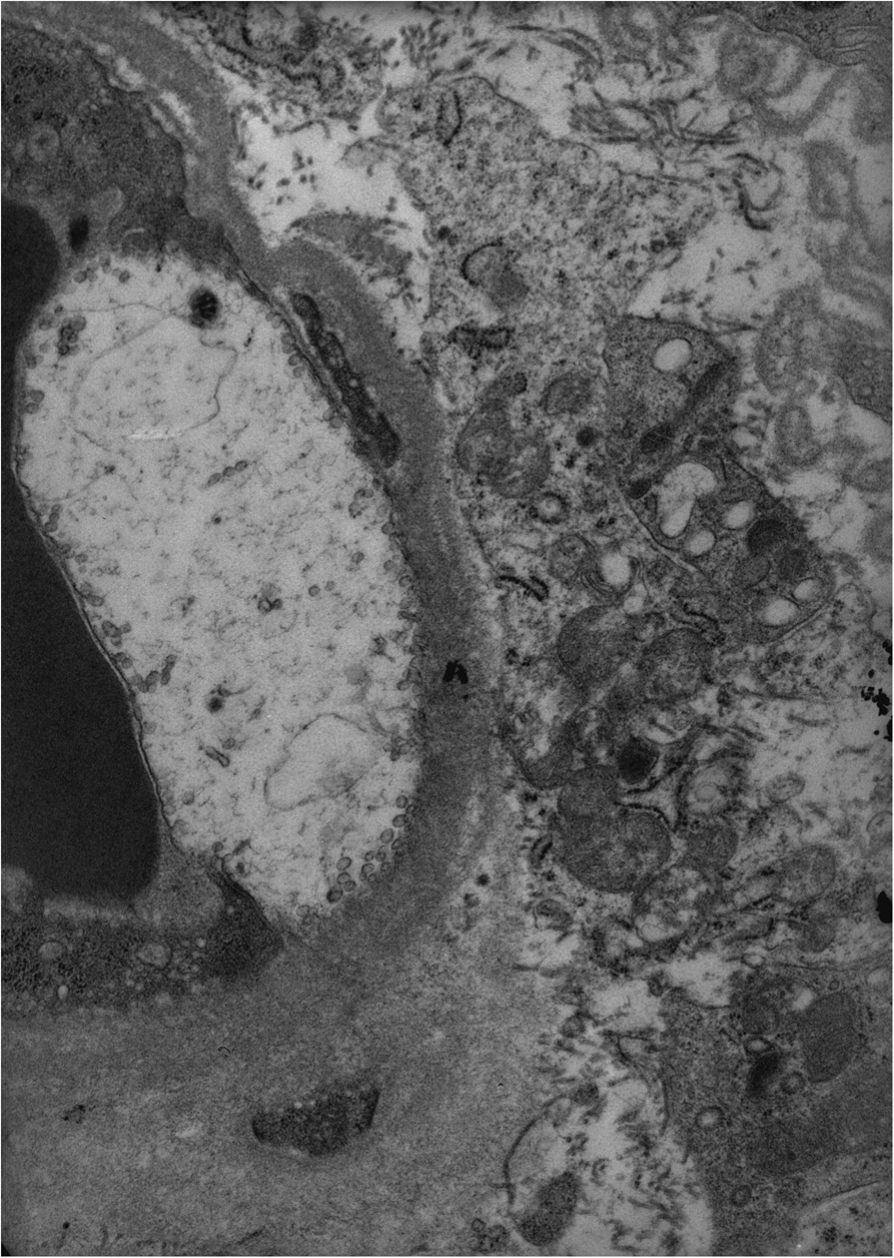
**Vastus lateralis muscle biopsy.** A swollen endothelial cell of an endomysial capillary, with loss of organelles and cytoplasmic material. Transmission electron microscopy, ×11,500.

**Figure 8:**
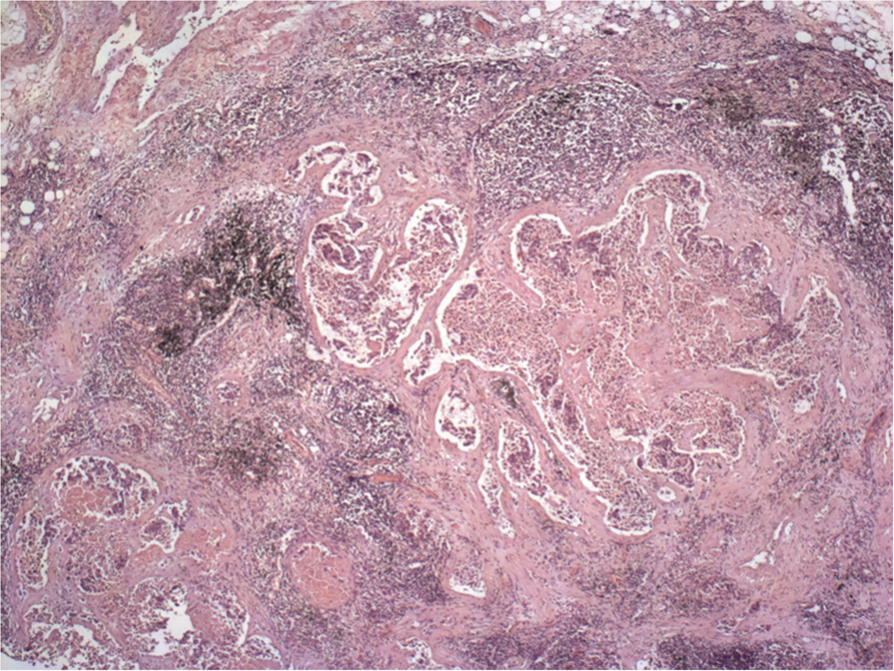
**Pulmonary hilar lymph node biopsy.** Regional lymphadenopathy (metastases). Hematoxylin and eosin staining, ×25.

**Figure 9:**
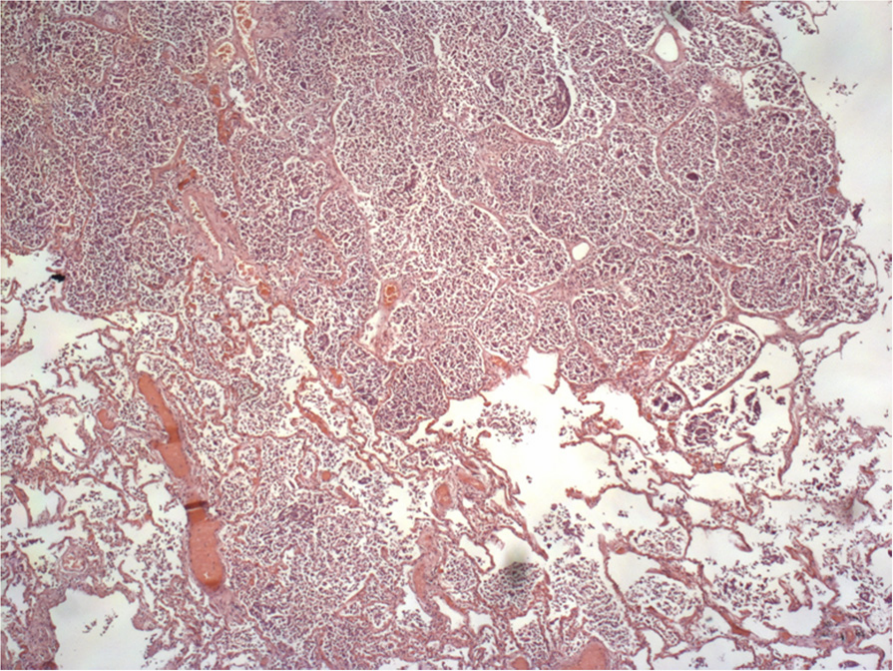
**Lung biopsy.** Adenocarcinoma with a necrotic component and surrounding edema. Bronchopneumonia and emphysema are observed. Hematoxylin and eosin staining, ×25.

Total body computed tomography showed a nodular mass in the apex of the right lung (25mm in diameter), enlarged lymph nodes below the tracheal bifurcation, and left lower lobe pneumonia. No metastatic lesions were found, and there were no abdominal or cerebral abnormalities. The patient was diagnosed with paraneoplastic necrotizing myopathy, and corticosteroid treatment was started (prednisolone 1.5mg/kg/day) together with antibiotics for pneumonia. There was no significant clinical improvement. The steroids were continued and the CK level slowly decreased. However, his weakness did not resolve, and he died of progressive respiratory failure 40 days after admission. Subsequent examination of resin-embedded biopsy specimens using the transmission electron microscope showed typical pipestem changes in the endomysial capillaries (thickening of the basal membrane and deposition of amorphous material) and swollen endothelial cells (Figures 6 and 7). Morphometric analysis of cryostat sections stained for endothelial marker CD31 confirmed decreased capillary density (148.2/mm^2^; normal range 393±29/mm^2^) (Figure 4). Post-mortem examination provided histological confirmation of a neoplasm of the right lung (2.5cm diameter). The tumor was a partly necrotic, poorly differentiated adenocarcinoma that infiltrated the lung parenchyma, with lymph node metastases. The post-mortem examination also revealed bilateral bronchopneumonia (Figures 8 and 9) and myocardial sclerosis. We did not treat our patient with chemotherapy or immunoglobulin because he had advanced cancer at the time of diagnosis and his prognosis was very poor. We did not test for anti-signal recognition particle (SRP).

### Search strategy

We searched PubMed using the terms “necrotizing myopathy” and “paraneoplastic necrotizing myopathy”.

## Discussion

We describe a case of paraneoplastic necrotizing myopathy in a patient with an undiagnosed lung adenocarcinoma. The patient had a history of ischemic heart disease, and was admitted to hospital for treatment of heart failure. His symptoms were initially attributed to a myocardial infarction because of his clinical history and electrocardiography and echocardiography findings, and the elevated serum muscle enzyme levels were attributed to myocardial ischemia. After admission, his heart failure resolved, but his muscle enzyme levels continued to increase. He developed symmetrical progressive proximal muscle weakness and was unable to mobilize. At this point, he already had advanced myopathy.

Initially, the diagnosis of necrotizing myopathy in a patient with ischemic heart disease and subacute onset of weakness with a high CK level led to investigation for drug-induced myopathy. The patient had never taken statins, proton pump inhibitors, or atypical antipsychotics that could have myotoxic effects. Of the drugs taken by the patient, to the best of our knowledge, only amiodarone is to be considered potentially myotoxic. Besides the more frequent peripheral neuropathy, amiodarone-associated myopathy has also been described (review by [13]), in most cases as a vacuolar autophagic myopathy with type II fiber atrophy, which was not seen in our patient. Although amiodarone-induced necrotizing myopathy has been previously reported, in the present case, peculiar pathological features, such as MAC capillary deposition and pipestem capillaries, are not consistent with amiodarone myopathy. Myotoxicity from salicylates has been reported anecdotally in one case of chronic abuse. Dermatomyositis may be associated with MAC deposition in capillaries, usually in a patchy pattern [6], but was ruled out because of the absence of skin lesions, perifascicular atrophy, and perivascular inflammation, and the rather diffuse MAC deposition pattern in capillaries. The pathological pattern also differed from that of polymyositis because of absence of significant endomysial infiltrates, lack of lymphohistiocytic invasion of non-necrotic fibers, and mild upregulation of MHC-I. As NAM has been reported in association with connective tissue disorders [3,5], and less frequently with cancer [2,14,15], immunologic and oncologic investigations were undertaken, leading to the diagnosis of lung cancer. Paraneoplastic necrotizing myopathies are rare and are known to be histopathologically heterogeneous, with myonecrosis ranging from occasional and scattered, to diffuse as in our patient [9]. MAC deposition has been reported in NAM, both in paraneoplastic [2,14] and non-neoplastic cases [2,7,14], but it may also be absent [9,14,15]. Capillary loss in association with MAC deposition, as was observed in our patient, was morphometrically assessed in one group of cases [14]. Few ultrastructural studies of microvessels in patients with NAM have been reported [2-4], and the pathogenic relationship between muscle necrosis and pipestem microangiopathy has yet to be elucidated. A subset of NAM is characterized by anti-SRP antibodies. SRP is a protein involved in protein trafficking in endoplasmic reticulum, and anti-SRP antibodies are classified as myositis-specific autoantibodies [6]. Occasional cases of paraneoplastic anti-SRP+ NAM have been reported [14,15]. No association between MAC deposition in capillaries and SRP antibodies has been detected, and there are no distinctive clinicopathological features that differentiate between SRP+ NAM and SRP– NAM. The role of anti-SRP antibodies in the pathogenesis of NAM is therefore unclear. Our patient was not tested for anti-SRP autoantibodies because testing was not available. Polyneuropathy may also occur in paraneoplastic NAM [14,15], in some cases associated with previous antiblastic treatment [8]. It is probable that our patient had a mild pre-existing sensory-motor axonal polyneuropathy, as mild fiber type grouping consistent with reinnervation was observed. The moderate upregulation of MHC-I with neoexpression on some non-necrotic fibers is a consistent feature of NAM, in contrast to the diffuse upregulation observed in polymyositis [9,11,14], and a cytokine response inhibiting upregulation of MHC-I in NAM has been hypothesized.

## Conclusion

This case illustrates that paraneoplastic necrotizing myopathy should be considered in elderly patients. Because the pathophysiology of paraneoplastic necrotizing myopathy is still unclear, there is no standard treatment regimen. Usually corticoid therapy and/or immunotherapy is suggested, but treatment of the underlying malignancy is the most important factor for achieving clinical improvement.

As necrotizing myopathy can worsen the prognosis of patients with cancer, early diagnosis and treatment are important. In patients who are unresponsive to corticosteroids, immunosuppressive therapy may be used (such as azathioprine, mycophenolate, methotrexate, or cyclosporine). Intravenous immunoglobulins are used as second-line therapy [14]. More recently, monoclonal antibodies such as rituximab have also been used for the treatment of myopathies including necrotizing myopathy [12].

## Consent

Our patient died during hospitalization and his next-of-kin was not traceable. Despite all reasonable efforts to find other family members, we were unable to obtain consent. We believe however that the patient would not have objected to publication of this case report, that all data was sufficiently anonymous, and that anyone who knew the patient would be unable to identify him from the published article.

## Abbreviations

CK: Creatine kinase; MAC: Complement membrane attack complex; MHC: Major histocompatibility complex; NAM: Necrotizing autoimmune myopathy; SRP: Signal recognition particle

## Competing interests

The authors declare that they have no competing interests.

## Authors’ contributions

AA and TA drafted the manuscript. AA, TA, SR, LF, VS, MF, and RN analyzed and interpreted the patient data regarding the myopathy and the lung tumor. NV performed the histological examination of the muscle biopsy specimen. All authors read and approved the final manuscript.
